# Genetics and Glaucoma: the state of the art

**DOI:** 10.3389/fmed.2023.1289952

**Published:** 2023-12-12

**Authors:** Sara Tirendi, Cinzia Domenicotti, Anna Maria Bassi, Stefania Vernazza

**Affiliations:** ^1^Department of Experimental Medicine, University of Genoa, Genoa, Italy; ^2^Inter-University Center for the Promotion of the 3Rs Principles in Teaching & Research (Centro 3R), Genoa, Italy

**Keywords:** genetics, Glaucoma, GWAS, MYOC, OPTN, CYP1B1

## Abstract

Glaucoma is the second leading cause of irreversible blindness worldwide. Although genetic background contributes differently to rare early-onset glaucoma (before age 40) or common adult-onset glaucoma, it is now considered an important factor in all major forms of the disease. Genetic and genomic studies, including GWAS, are contributing to identifying novel loci associated with glaucoma or to endophenotypes across ancestries to enrich the knowledge about glaucoma genetic susceptibility. Moreover, new high-throughput functional genomics contributes to defining the relevance of genetic results in the biological pathways and processes involved in glaucoma pathogenesis. Such studies are expected to advance significantly our understanding of glaucoma’s genetic basis and provide new druggable targets to treat glaucoma. This review gives an overview of the role of genetics in the pathogenesis or risk of glaucoma.

## 1 Introduction

Glaucoma is the second leading cause of blindness worldwide, and it is estimated that its incidence will increase in the next 20 years (1–3). The term glaucoma brings together a heterogeneous group of neurodegenerative diseases characterized by Retinal Ganglion Cell (RGC) degeneration as well as typical morphological changes in the Optic Nerve Head (ONH), i.e., the narrowing of the neuroretinal rim, the enlargement of the excavation, and the loss of prelaminar neural tissue (4, 5).

In general, all glaucoma forms are categorized into primary and secondary forms, according to their etiology. Primary glaucoma is defined as an isolated, idiopathic disease of the anterior chamber of the eye and the optic nerve whereas the secondary one is characteristic of specific syndromic frameworks (e.g., nail patella, exfoliation syndrome, aniridia), or associated with predisposing events, including systemic diseases (e.g., diabetes) and pharmacological treatment.

Primary glaucoma is further distinguished into three broad categories, based on the anatomy of the anterior chamber drainage angle, aqueous dynamics, and age at onset: (i) Primary Open Angle Glaucoma (POAG), (ii) Primary congenital glaucoma (PCG), and (iii) Primary Angle Closure Glaucoma (PACG) (3, 6).

Primary open angle glaucoma is responsible for most cases of adult-onset glaucoma (i.e., individuals aged over 40 years) in Africans, Latinos, Americans, and European populations (7, 8). However, POAG, to a lesser extent, may occur in young subjects ranging from 3 years old and 40 years of age, as in the case of Juvenile Open Angle Glaucoma (JOAG).

Primary congenital glaucoma is a rare form of glaucoma in infancy (before the age of 3 years) characterized by aqueous humor drainage obstruction due to an upstream non-development of neural crest-derived tissues, which results in developmental anomaly at the angle of the anterior chamber (9).

Finally, PACG is responsible for most cases of glaucoma-induced bilateral blindness in populations, such as Asians (10). In such glaucoma, the anatomic narrow-angle leads to trabecular meshwork (TM) obstruction and, consequently, to peripheral anterior synechiae, elevated intraocular pressure IOP, iris whirling or sectoral atrophy, and excessive pigment deposition on the trabecular surface (10–13).

Glaucoma can occur at all ages exhibiting a classic (Mendelian) inheritance pattern (i.e., onset before the age of 40) (14, 15) or a complex inheritance pattern [i.e., onset after the age of (16, 17)]. Therefore, genetic background is considered a precondition that can directly cause or contribute to glaucoma. This present review aims to summarize the main achievement in genetic studies of glaucoma, mainly focusing on glaucoma-causing genes.

## 2 Methods

The data in this present comprehensive review were collected using two different searches: PubMed^1^ and the online database: genome aggregation database,^2^ and the NHGRI-EBI Catalog of Human Genome-Wide Association Studies.^3^ The search of the references using PubMed identified a total of 157 hits with no time frame relative to keywords such as “Genetic of glaucoma” “POAG guidelines,” “JOAG management,” “MYOC mutations in glaucoma,” “CYP1B1 in glaucoma,” “PACG guidelines,” “clinical and genetic update of glaucoma,” “candidate genes involved in glaucoma.” Thus, the combined information obtained from articles relevant to the subject matter and the online database has represented the basis for writing the review.

## 3 Results

### 3.1 Primary open angle glaucoma

Primary open angle glaucoma is defined as a progressive chronic bilateral or asymmetric ocular disease (18). Clinical evidence in addition to visual field defects and the age of patients (i.e., > the 40s) includes structural abnormalities to the optic disk (OD) rim or parapapillary retinal nerve fiber layer (RNFL) (19). The severity of POAG damage can be distinguished as mild, moderate, and severe based on the presence or not of possible changes in the optic disk, RNFL damage, and degree of visual field abnormalities.

Although it is not an effective treatment for all patients, IOP management is the only clinically addressable risk factor to prevent or delay POAG progression (3). Primary open angle glaucoma guidelines (20) suggest the use of topical medications (e.g., Prostaglandin analogs, beta-blockers, alpha_2_ adrenergic agonists, parasympathomimetic, and topical and oral carbonic anhydrase inhibitors) as the first line of treatment to reach the target pressure. However, since treatment regimens require daily applications to control IOP, noncompliance, undesirable adverse effects, and cost limit their effectiveness (21, 22). Laser trabeculoplasty and glaucoma surgery (e.g., trabeculectomy, aqueous shunts, non-penetrating glaucoma surgery, micro-invasive glaucoma surgery, and so on…) are considered for further IOP reduction in eyes with inadequate initial responses (23).

Adult-onset POAG is a complex disease often caused by an intricate interplay at molecular levels between predisposing genes and evidence-based risk factors (e.g., ethnicity, gender, aging, IOP, central corneal thickness, migraine headache and peripheral vasospasm, myopia, systemic diseases, steroid responsiveness, positive history of glaucoma) (3, 24, 25). Primary open angle glaucoma may be inherited as a Mendelian trait, as in the case of causative mutations in genes such as the myocilin (MYOC), optineurin (OPTN), and TANK-binding kinase 1 (TBK1) (26, 27), where other genes or environmental factors play little role (*See below*).

Generally, POAG is unsuitable for linkage studies because they require many living POAG-affected relatives and this, for obvious reasons, is not always possible. Thus, highlighting an inheritance pattern for POAG represents a real challenge compared to the childhood-onset glaucomas (24, 28–31).

Genome-Wide Association Studies (GWAS) provide information about the common POAG genetic variants, i.e., Single Nucleotide Polymorphisms (SNPs), that are found among populations of diverse ancestry (Table 1). In this regard, recurring SNPs may constitute a quantifiable relationship between the genetic variability of POAG and its clinical manifestation (endophenotypes) (30, 41, 42). However, SNPs have limited predictive value in terms of risk assessment and prognosis of POAG due to its complex inheritance pattern (43).

**Table 1:** List of some known SNPs identified in different glaucoma cohorts (source: GWAS Catalog).

Variant and risk allele	Location	Mapped gene(s)	Alleles	Ancestry	Ref.
rs2041895-C	12:106956310	TMEM263	C/A/G/T	European	(32)
rs1333037-T	9:22040766	CDKN2B-AS1	C/T	European, Sub-Saharan African, East Asian, South Asian, African unspecified, Afro-Caribbean, Hispanic or Latin American	(32–34)
rs4657477-T	1:165766938	TMCO1	C/T	Sub-Saharan African East Asian European	(34)
rs2472494-C rs2472494-T	9:104933258	CT70, ABCA1	T/A/C	East Asian Sub-Saharan African European African American or Afro-Caribbean	(34–36)
rs57552558-T rs62283811-T rs62283809-T	3:172114745 3:172103100 3:172102421	FNDC3B	C/A/T C/A/G/T T/G	European Sub-Saharan African East Asian	(34)
rs1536907-G rs11792928-T rs10448285-T rs3829849-T	9:126620281 9:126639271 9:126634735 9:126628521	LMX1B	A/G/T C/T C/T C/T	European Hispanic or Latin American Other admixed ancestry African American or Afro-Caribbean South Asian African unspecified East Asian	(33, 34)
rs61870251-A	10:124595846	LHPP	A/C	East Asian	(34)
rs1550437-T rs8034017-A	15:73928957 15:73929702	LOXL1	C/T A/G	East Asian Sub-Saharan African European	(34)
rs7588567-G	2:133605461	RN7SKP93	T/A/C/G	East Asian	(24)
rs4236601-A	7:116522675	CAV2, CAV1	G/A	European	(37, 38)
rs62391632-T rs11969985-G rs9392348-G rs12664587-A	6:1957768 6:1922673 6:1989370 6:1948469	GMDS	G/T G/A G/A A/T	Sub-Saharan African East Asian European	(34, 38)
rs1647381-G	15:56794001	ZNF280D	C/G	European Hispanic or Latin American Other admixed ancestry African American or Afro-Caribbean South Asian African unspecified East Asian	(33)
rs33912345-A	14:60509819	C14orf39, SIX6	C/A/G	European East Asian Sub-Saharan African	(32, 34)
rs9913911-A rs12150284-T	17:10127866 17:10127773	GAS7	A/G C/A/G/T	African American or Afro-Caribbean European Hispanic or Latin American East Asian Other admixed ancestry South Asian African unspecified Sub-Saharan African	(33, 34, 39, 40)
rs7137828-T	12:111494996	ATXN2	C/A/T	European East Asian	(32)

#### 3.1.1 Juvenile open angle glaucoma

Juvenile open angle glaucoma, as above mentioned, is an uncommon subset (0,38,100,000) of POAG that affects individuals during childhood or early adulthood.

Generally, JOAG is inherited in an autosomal dominant fashion in individuals with a strong family history of glaucoma. Nevertheless, it has been shown that in Iranian and Saudi Arabian populations, it occurs as an autosomal recessive or sporadic disease (17, 44–46). Therefore, genetically it is possible to identify different pathological mutations, including myocilin (MYOC), Cytochrome P450 subtype 1 Polypeptide 1 (*CYP1B1*), Optineurin (*OPTN*), WD Repeat domain 36 (*WDR36*), Neurotrophin 4 (*NTF4*), Latent Transforming growth factor-beta-binding Protein 2 (*LTBP2*), and Ankyrin repeat and SOCS-Box containing 10 (*ASB10*) (47–50). Recently, also the rare biallelic mutation CPAMD8 in both JOAG and PCG was identified as a unique form of autosomal recessive anterior segment dysgenesis with a variable range of clinical phenotypes (51).

In most cases, JOAG is characterized by severe IOP elevation (>40 mmHg) due to immaturity of the conventional outflow pathway, and thick corneas. However, the extent of clinical manifestations, including the IOP elevation, degree of goniodysgenesis, and the inheritance pattern, as well as the age at onset, are subject to individual variations. In this regard, four phenotype groups have been identified to classify affected individuals based on the age of onset, highest untreated IOP, gonioscopic findings, and iris features (52) (Table 2).

**Table 2:** Four phenotypic groups of JOAG.

	Group 1	Group 2	Group 3	Group 4
Iris crypts	Normal	Normal	Normal	Absent prominent
Angle	Normal	Featureless angle	Prominent iris processes or high iris insertion	Prominent iris processes or high iris insertion
Age of onset	28 ± 9.2 years	24 ± 9.6 years	26 ± 7.8 years	27.4 ± 9.2 years
IOP	36 ± 11 mmHg	38.8 ± 12.8 mmHg	41.3 ± 12.7 mmHg	41.3 ± 12.7 mmHg

Recent evidence also reported a JOAG subtype, i.e., Juvenile normal tension glaucoma (JNTG), in which glaucomatous damage occurs at normal-range IOP (≤ 21 mmHg) (53).

Juvenile open angle glaucoma differs from other early-onset glaucoma forms (e.g., congenital glaucoma) by the absence of buphthalmos, megalocornea, Haab’s striae, and ocular or other systemic developmental anomalies (17, 54).

Juvenile open angle glaucoma patients are often asymptomatic until an advanced stage, therefore, a prompt diagnosis is not always possible. When JOAG becomes symptomatic results in blurred vision, eye pain, decreased visual acuity, tearing, blinking, and glare (55). As known, an early JOAG diagnosis increases the chance of preserving vision and preventing permanent visual field loss. Surgery (i.e., trabeculectomy, external trabeculectomy, goniotomy, drainage implants, gonioscopy-assisted transluminal trabeculectomy, and so on…) is often required since the affected individuals are refractory to the standard primary drug approaches to manage the IOP (e.g., β blockers, topical carbonic anhydrase inhibitor, prostaglandin analogs, α-adrenergic agonists) (56, 57).

#### 3.1.2 Myocilin

The *MYOC* gene, also known as Trabecular meshwork Inducible Glucocorticoid Response (TIGR), maps to the GLC1A (Glaucoma 1A) locus at chromosome 1q21– q31. It consists of three exons: the first encodes for amino-terminal region of the protein containing a peptide signal sequence and a leucine zipper-like motif (i.e., where the myocilin-myocilin interactions take place) with periodic arginine and leucine repeats arranged along an α-helix; the second encodes for the protein central region; the third encodes for the carboxyl-terminal half of myocilin, the olfactomedin (OLF)-domain, which includes the majority of the identified disease causing variants (58).

Myocilin encodes the myocilin glycoprotein expressed in several ocular tissues, such as the cornea, iris, ciliary body, retinal epithelium, and TM. Myocilin mutations can be responsible for the improper function of the eye tissue where the protein is commonly highly expressed, e.g., TM (59). Moreover, myocilin is found also in non-ocular tissues, such as the heart tissue and skeletal muscle but there is no evidence of its involvement in systemic disease (60, 61).

Myocilin in its wild-type (wt) state is a ubiquitous protein whose function in the eye has not been determined yet. One suggestion is that myocilin contributes to the maintenance of IOP homeostasis, although it has not been confirmed due to conflicting and contradictory data. While some studies report that myocilin up-regulation is responsible for IOP elevation (62, 63), other ones report that neither its overexpression nor its haploinsufficiency is the primary mechanism for glaucoma phenotype. (31, 64, 65). This latter evidence has challenged the pathological role of myocilin in promoting steroid-induced glaucoma (~40% of cases) after long-term glucocorticoid treatment (e.g., dexamethasone) (66). In this regard, previous studies on steroid-induced myocilin overexpression excluded its role in both ocular hypertension and endoplasmic reticulum (ER) stress (67, 68). However, a myocilin stress-related function in TM has been suggested because stressful stimuli, such as hydrogen peroxide and elevated IOP, increase its expression (69, 70).

The first evidence that showed a correlation between POAG and mutant myocilin date from 1997 (71). Linkage studies have shown that MYOC mutations, accounting for 8–34% of JOAG, 2–4% of adult-onset POAG cases, and in part for NTG, are mainly inherited in an autosomal dominant manner with incomplete penetrance (72). In this regard, disease severity varies between individuals due to a strong genotype–phenotype correlation (73). A meta-analysis of the penetrance of MYOC mutation revealed that except for a few variants (e.g., Pro370Leu, Thr377Arg, Asp380Ala, and Asn480Lys) having a stable penetrance, the penetrance of MYOC mutations can be influenced by factors such as aging, and ethnicity (74).

Under physiological conditions, wt myocilin after intracellular endoproteolytic processing, produces two fragments, i.e., the 35kD fragment containing the C-terminal OLF-like domain and the 20kD fragment containing the N-terminal leucine zipper-like domain (75). However, only the 35kD fragment is co-secreted with non-processed protein leading to assume that it may play a role in the regulation of the myocilin extracellular activity (76, 77). The best known-myocilin-induced disease phenotype is characterized by an intracellular abnormal buildup of myocilin in TM (26, 78, 79), resulting in a toxic gain-of-function disease mechanism. Indeed, most of the myocilin-associated glaucoma mutations are missense clustered in the OLF domain (Table 3). These mutations by inducing defects in myocilin stability, promote the mutant myocilin retention into the endoplasmic reticulum (ER) of TM cells with toxic consequences, such as ER stress, cytotoxicity, and TM apoptosis, which in turn affect the IOP control (61, 75, 91–93). However, also nonsense mutations are involved in about 5.7% of JOAG and adult-onset POAG cases. Notably, the nonsense mutation Gln368Stop is responsible for 40% of High Tension Glaucoma (HTG) (91, 94, 95).

**Table 3:** List of some pathogenic/likely pathogenic glaucoma-associated myocilin missense mutations.

SNP	Placement		Position	Ref.
	MYOC transcript	Myocilin		
	NM_000261.2:c.1440C > G	NP_000252.1:p.Asn480Lys	1: 171636000	(80, 81)
rs74315332	NM_000261.2:c.1440C>A	NP_000252.1:p.Asn480Lys	1: 171636000	(81)
rs74315338	NM_000261.2:c.1297T>C	NP_000252.1:p.Cys433Arg	1: 171636143	(82)
rs74315328	NM_000261.2:c.1309T>C	NP_000252.1:p.Tyr437His	1: 171636131	(81, 83, 84)
rs74315335	NM_000261.2:c.1010A>G	NP_000252.1:p.Gln337Arg	1: 171636430	(85)
	NM_000261.2:c.1139A>C	NP_000252.1:p.Asp380Ala	1: 171636301	(80, 84, 86, 87)
rs74315329	NM_000261.2:c.1102C>T	NP_000252.1:p.Gln368Ter	1: 171636338 non sense	(88–90)
rs74315334	NM_000261.2:c.1099G>A	NP_000252.1:p.Gly367Arg	1: 171636341	(81)
	NM_000261.2:c.1130C>T	NP_000252.1:p.Thr377Met	1: 171636310	(88)
rs74315330	NM_000261.2:c.1109C>T	NP_000252.1:p.Pro370Leu	1: 171636331	(81, 83, 84)

Source: gnomAD browser.

#### 3.1.3 Familial normal tension glaucoma

Normal-tension glaucoma (NTG) is an OAG characterized by an IOP within a statistically normal range (≤21 mmHg). The NTG pattern of damage in the optic disk, retinal nerve fiber layer, and visual field significantly differs from that found in POAG, suggesting that the optic neuropathy is due to other mechanisms rather than elevating IOP (96). In this regard, a higher sensitivity to normal pressure, vascular dysregulation, an abnormally high translaminar pressure gradient, and a low cerebrospinal fluid pressure in the optic nerve sheath compartment, have been proposed to contribute to NTG pathogenesis (97).

Generally, NTG has a complex genetic basis, but the analyses of several pedigrees showed that about 2% of NTG forms (i.e., Familial NTG) are caused by a single-gene mutation. NTG genetic survey improved thanks to the GWAS since have identified several susceptible loci contributing to NTG on chromosomes 9p2, 2p21, 7q31, 1q24, and 6p21.1-p12.1 (37, 43, 96, 98).

Familial NTG refers to an early onset (at 20–35 years old) bilateral disease with variable severity (99). Optineurin, MYOC, and TBK1 are indicated as NTG-causing genes, according to the Mendelian autosomal dominant inheritance pattern.

#### 3.1.4 Optineurin

The human *OPTN* gene, located on the short arm of chromosome 10 (10q13), encodes for optineurin, which is a multifunctional protein ubiquitously expressed in many tissues (e.g., the heart, brain, liver, skeletal muscle, kidney, pancreas, and the eye). In the eye, optineurin is expressed in the trabecular meshwork, non-pigmented ciliary epithelium, and remarkably in the retina (100). The well-characterized optineurin multiple cellular functions include selective autophagy (i.e., mitophagy, aggrephagy, xenophagy) through ubiquitin-signaling, NF-κB regulation, membrane trafficking, exocytosis, vesicle transport, transcriptional activation, and reorganizing of actin and microtubules. All these molecular functions arise from the interaction with a number of proteins, such as Rab8, huntingtin (Htt), transcription factor IIIA, myosin VI, and TANK binding protein 1 (TBK1) (101–103).

The optineurin-mediated autophagy-dysfunction by either overstimulation or by defects that block its function has attracted considerable attention due to its involvement in a variety of neurodegenerative diseases (glaucoma, amyotrophic lateral sclerosis, Parkinson’s disease, Huntington’s disease), inflammatory diseases (Crohn’s disease, acute liver failure, rheumatoid arthritis) cancer, and nephropathy (104). Notably, the autophagy-mediated RGC death in POAG, also known as autophagic cell death, seems to be promoted by an excess of catabolic activity induced by chronic IOP elevation (105) or alterations in OPTN and TBK1 (106). Under physiological conditions, OPTN to link structures targeted for elimination with proteins of an assembling autophagosome must be phosphorylated by TBK1. However, POAG-associated OPTN mutations may increase the autophagy activation by promoting OPTN phosphorylation via TBK1. Moreover, other OPTN functions may be altered by specific mutations (*see below*). On the other hand, the gain-of-function mutation and copy number variation (CNV) of the TBK1 gene have been proposed as possible Normal Tension Glaucoma (NTG, a POAG subtype) causing mechanism (107, 108).

Rezaie et al. (109) described four sequence alterations in OPTN involved in autosomal dominant adult-onset glaucoma cases, i.e., Glu50Lys, Arg545Gnl, 691_692insAG, and Met98Lys. Most of the affected families harboring these OPTN alterations had normal IOP, and only a small portion had high IOP. Among the OPTN polymorphisms, Glu50Lys, which is located within a putative bZIP motif, is the most recurrent likely disease-causing variant associated with hereditary NTG in Caucasian and Hispanic individuals. The Glu50Lys-induced functional alterations leading to RGC apoptosis are probably related to both autophagy and trafficking dysfunction and an increase in oxidative stress (110, 111).

Arg545Gnl may be considered a disease-causing variant only in Asian populations because this role was not shown in other studies (112). 691_692insAG variant was described as a dominant mutation responsible for familial POAG in Japan (109) and Eastern Europe (112). Met98Lys was initially proposed as an attributable risk factor for familial and sporadic POAG cases but due to conflicting results among diverse populations, it was not confirmed (113–115).

Interesting, semiquantitative, hierarchical evidence-based rules for locus interpretation (Sherloc), which are a refinement of the American College of Medical Genetics and Genomics–Association for Molecular Pathology (ACMG–AMP) guidelines, are providing an accurate clinical prediction of the functional consequences of OPTN mutations (116). The algorithms used to predict the effect of sequence change suggest that variants affecting protein sequence (e.g., missense mutation), its function (e.g., splicing mutation), or its abundance (e.g., frameshift or nonsense mutations) are presumed more likely to be deleterious (Please visit the National Library of Medicine for more information).^4^ Further studies on larger populations are required to confirm the incrimination of OPTN in POAG, to discriminate OPTN mutations causing POAG from those acting only as risk factors, and to ascertain the rate of loss-of-function OPTN variants in different populations.

### 3.2 PGC

Primary congenital glaucoma is considered a non-syndromic glaucoma form characterized by a childhood onset with variable incidence across countries and ethnic groups, but it is most widespread in countries where consanguineous marriages abound (e.g., Middle East) (117, 118).

This pediatric glaucoma occurs at birth or within the first 3 years of life, and the clinical outcomes vary depending on whether the neonatal/newborn-onset PGC (within a month after birth) or late-onset-PGC (after 2 years of age). Indeed, neonatal PGC has a poor visual prognosis compared to that diagnosis later, especially if accompanied by amblyopia (119, 120). Moreover, other elements strongly associated with poor long-term visual outcomes are unilateral disease, multiple surgeries, poor vision at diagnosis, and ocular co-morbidities.

The main mechanisms involved in PCG are related to trabecular dysgenesis due to mutations affecting normal TM development with or without iridodysgenesis and maldeveloped trabecular angle meshwork with or without dysgenesis of the Schlemm’s canal (121–123). Moreover, histopathological findings of the anterior chamber angle of PCG eyes reveal an immature anatomic relationship involving iris, TM, and ciliary body (119, 124, 125). Clinical features include high IOP, an increase in horizontal corneal diameter, the presence of corneal edema, Haab’s striae, the pupillary reflex, refraction, axial length, and keratometry (124).

High IOP and its fluctuations are managed through multiple or single surgical drainage procedures (e.g., goniotomy and trabeculotomy ab externo) aimed at targeting the abnormal trabecular angle. Topical hypotensive medications could be used post-op (126).

Genetically, PCG is considered heterogeneous. 10–40% of affected individuals show several pathogenetic variants of CYP1B1 or LTBP2 inherited in an autosomal recessive manner and with varying degrees of penetrance (127). Moreover, also autosomal dominant and pseudo-dominant inheritance patterns have been described as well (117, 128, 129).

Genetic linkage analysis has identified four chromosomal loci of recessively inherited PCG, namely GLC3A, GLC3B, GLC3C, and GLC3D. Among these, GLC3A on chromosome 2p22-p21, which harbors the CYP1B1 gene, is considered the most representative of the severe phenotypes (123) (Table 4).

**Table 4:** List of some pathogenic/likely pathogenic glaucoma-associated CYP1B1 missense mutations (Source: gnomAD browser).

	Placement			
dsSNP	CYP1B1 transcript	Cytochrome P450 1B1	Position	Ref.
rs1682415237	NM_000104.4:c.1460 T > C	NP_000095.2:p.Leu487Pro	2:38070894	(130)
rs28936701	NM_000104.4:c.1405C > T	NP_000095.2:p.Arg469Trp	2:38070949	(131, 132)
rs72549376	NM_000104.4:c.1331G > A	NP_000095.2:p.Arg444Gln	2:38071023	(133)
rs56175199	NM_000104.4:c.1310C > T	NP_000095.2:p.Pro437Leu	2: 38071044	(134)
rs104893629	NM_000104.4:c.1267A > T	NP_000095.2:p.Asn423Tyr	2:38071087	(135)
rs148542782	NM_000104.4:c.1168C > A	NP_000095.2:p.Arg390Ser	2:38071186	(136)
rs55989760	NM_000104.4:c.1159G > A	NP_000095.2:p.Glu387Lys	2: 38071195	(137, 138)
rs56175199	NM_000104.4:c.1310C > T	NP_000095.2:p.Pro437Leu	2:38071044	(134)
rs55771538	NM_000101.4:c.1093G > T	NP_000095.2:p.Gly365Trp	2:38071261	(85)
rs72549379	NM_000101.4:c.1090G > A	NP_000095.2:p.Val364Met	2:38071264	(139, 140)
rs52976968	NM_000101.4:c.578C > T	NP_000095.2:p.Pro193Leu	2:38074811	(141–143)
rs72481807	NM_000101.4:c.517G > A	NP_000095.2:p.Glu173Lys	2:38074872	(133, 144, 145)
rs28936700	NM_000101.4:c.182G > A	NP_000095.2:p.Gly61Glu	2:38075207	(131, 146, 147)
rs72549389	NM_000101.4:c.2 T > C	NP_000095.2:p.Met1Thr	2:38075387	(148, 149)
rs757520959	NM_000101.4:c.1A > G	NP_000095.2:p.Met1Val	2:38075388	

#### 3.2.1 Cytochrome P450 subtype 1 polypeptide 1

Cytochrome P450 subtype 1 Polypeptide 1, belonging to the cytochrome P450 (CYP450) superfamily, is an enzyme mainly involved in oxidative metabolic activation (e.g., synthesis of retinoic acid) and detoxification of many compounds (e.g., several polycyclic aromatic hydrocarbons, endogenous steroids) (150).

Human *Cyp1b1* consists of three exons and two introns, where the coding region is located within the second and the third exons. It is expressed constitutively in many parenchymal and stromal tissues, including the liver, brain, kidney, prostate, breast, cervix, uterus, ovary, lymph nodes, heart, placenta, lung, skeletal muscle, and eye (85, 151).

The role of CYP1B1 in PCG and JOAG is not clear, but it has been hypothesized that it plays a metabolic role in eye development and function, as well as cellular redox homeostasis maintenance (152–154). Therefore, the most accepted theory to explain the link between CYP1B1 mutation and PCG holds that mutated CYP1B1 is the leading cause of maladapted and dysfunctional TM due to defects in embryonic neural crest cell migration during development. This condition, in turn, leads to increased IOP, compression of the optic nerve, and blindness, if not treated surgically (155). Moreover, mutations in the *Cyp1b1* gene, in addition to being strongly related to the pathophysiology of PCG, are associated with the most severe disease outcomes. Notably, a genotype–phenotype study in a large cohort of Moroccan PCG reports that both patients carrying CYP1B1 mutation and those with double CYP1B1 null alleles showed the most severe phenotype when compared with patients genetically negative (156).

Most of the CYP1B1 mutations responsible for a maldeveloped and dysfunctional TM are missense (Table 5), but how they affect disease phenotype and their frequency among different populations is still under investigation (152, 155). Recently, “*in silico*” methods (supported by databases, network analysis, and machine learning approaches) were used to identify possible high-risk deleterious variations by predicting the functional importance (neutral or deleterious) of the altered amino acid substitution on protein stability and the resulting phenotypic characteristics (e.g., disruption of the structural conformation), considering the allele frequency of variants (157). Based on such approaches, three novel missense mutations, i.e., Leu487Pro, Leu177Arg and Trp434Arg, and a transversion mutation Asp374Glu were identified in consanguineous Pakistani families as impacting the CYP1B1 protein stability, structure, and function (158). The results obtained from computational tools, functional assays, segregation analysis, genetic studies, and epidemiological data could potentially be helpful for the understanding and management of PCG.

**Table 5:** SNPs of PACG susceptibility loci identified in different glaucoma cohorts (source: GWAS Catalog).

Variant and risk allele	Location	Mapped gene(s)	Alleles	Study accession
rs3816415-A	7:37948709	SFRP4, EPDR1	G/A	GCST003467
rs736893-G	9:4217028	GLIS3	G/A/C	GCST003467
rs3739821-G	9:127940198	FAM102A, DPM2	A/G	GCST003467
rs7494379-G	14:52944673	FERMT2	C/G/T	GCST003467
rs3753841-G	1:102914362	COL11A1	G/A/C	GCST003467
rs1015213-A	8:51974981	PCMTD1	C/T	GCST003467
rs11024102-G	11:16987058	PLEKHA7	T/A/C	GCST003467

### 3.3 Primary angle closure glaucoma

Primary angle closure glaucoma (PACG) is characterized by at least 180° of iridotrabecular contact, resulting from mechanical obstruction of the TM in the anterior chamber angle and glaucomatous optic neuropathy due to elevated IOP. PACG pathogenesis can be explained as a pupillary block, which is the most common one, plateau iris or peripheral iris crowding, and multiple mechanism patterns, according to clinical phenotype, anatomic configurations, etiology and natural history (159).

From a clinical point of view, PACG is classified into acute, sub-acute, and chronic angle-closure glaucoma, based on the timing of onset, symptoms, and clinical findings. However, is not unusual to observe a combination of these clinical types in patients. Indeed, acute/subacute angle-closure glaucoma may progress to a chronic stage if it does not remit spontaneously or under medical intervention or *vice-versa*.

In acute glaucoma, the sudden elevation of IOP (usually more than 50 mmHg) leads to severe symptoms, including pressure-induced corneal edema (i.e., blurred vision and halos around lights), mid-dilated pupil, lens opacity (glaucomflecken), vascular congestion, headache, eye pain, and redness (160). The subacute presentation, which may occur before the acute attack or before peripheral anterior synechiae (in the case of chronic IOP elevation), can be asymptomatic or show mild prodromal symptoms, such as headache, blurred vision, and halos, coinciding with IOP spikes (161). Finally, in chronic angle-closure glaucoma, IOP is usually chronically raised and generally, no symptoms are experienced when vision loss occurs. The closure of the angle is due to peripheral anterior synechiae (more than 180 degrees), which occurs as a consequence of recurrent pupillary block or “creeping” angle closure (162, 163). Clinical goals of PACG management are reversing or preventing the angle-closure process, controlling IOP through topical ocular hypotensive agents, laser peripheral iridotomy or refractive lensectomy, and rescuing optic nerve from damage (164).

Diagnosis of PACG is associated with its natural course and is often delayed until an advanced phenotypic stage, i.e., when the patient has chronic visual loss or acute angle closure. Several risk factors, such as aging, female gender, Inuit, East Asian, and South Indians ethnicity, anatomical features (e.g., shallow anterior chamber depth, short axial length, thick and anteriorly positioned lens, plateau iris configuration, and hyperopia), family history (i.e., first-degree relatives), and environmental factors, are involved in promotion of the disease progression (165–167).

PACG is characterized by a complex inheritance, thus to increase knowledge of the common variant contribution, GWAS have genotyped eight SNPs of PACG susceptibility loci for the common adult-onset form of PACG within PLEKHA7 (pleckstrin homology domain containing A7), COL11A1 (collagen type XI alpha 1 chain), PCMTD1-ST18 [Protein-L-Isoaspartate (D-Aspartate) O-Methyltransferase Domain Containing] 1-ST18 C2H2C-type zinc finger transcription factor (PCMTD1-ST18), EPDR1 (ependymin related 1), CHAT (choline O-acetyltransferase), GLIS3 (GLIS family zinc finger 3), FERMT2 (fermitin family member 2), DPM2-FAM102A (dolichyl-phosphate mannosyltransferase subunit 2, regulatory and family sequence similarity 102 member A, respectively; Table 5) (14). However, these SNPs explain only 2% of the genetic variance in PACG. Thus, they could not be considered potential universal gene-dependent risk factors for PACG pathogenesis since the genotype distribution and allele frequency varies among populations of different ancestry. (168, 169).

## 4 Conclusion

Although impressive advancements in glaucoma study and treatment are progressing, it still represents the second leading cause of blindness worldwide. Only a small portion of rare early-onset glaucomas (before age 40) show Mendelian inheritance, while the common adult-onset forms are characterized by a complex inheritance.

GWAS studies aim to identify either genetic variants associated with disease or endophenotypes across ancestries and important functional insights underlying biological mechanisms of glaucoma pathogenesis. GWAS results are clinically important to identify subjects with an increased risk of the disease and they can be the starting point for studying the contribution of protein-changing, coding sequence genetic variants. Thus, next-generation sequencing technologies coupled with high throughput functional genomics can enrich our understanding of the disease pathophysiological mechanisms by identifying novel disease-associated or causative genetic variants for predicting glaucoma risk. However, although gene-based screening can be useful for genetic counseling and treatment and surveillance plans in families with a history of early-onset glaucoma, a limitation is a relatively low diagnostic yield after testing for the genes currently known (170). Moreover, gene-based screening for adult-onset glaucoma seems to be accurate when comprises a large number of risk alleles.

The study of the complex genetic background in glaucoma is in the making but in the future, the hope is that it will become a tool to assess personal risk for the disease and facilitate novel therapeutic strategies.

## Author contributions

ST: Conceptualization, Writing – original draft, Writing – review & editing. CD: Supervision, Writing – review & editing. AB: Supervision, Writing – review & editing. SV: Conceptualization, Supervision, Writing – original draft, Writing – review & editing.

## Footnotes

^1^ https://pubmed.ncbi.nlm.nih.gov/

^2^ https://gnomad.broadinstitute.org/

^3^ https://www.ebi.ac.uk/gwas/

^4^ https://www.ncbi.nlm.nih.gov/clinvar/?term=OPTN%5Bgene%5D&redir=gene
